# Moxidectin is a candidate for use as an *in vivo* internal standard in pharmacokinetic studies, as demonstrated with use in simultaneous tissue cage and ultrafiltration fluid collection

**DOI:** 10.3389/fvets.2024.1332974

**Published:** 2024-01-16

**Authors:** Richard Munn, Ted Whittem

**Affiliations:** ^1^Melbourne Veterinary School, Faculty of Science, University of Melbourne, Werribee, VIC, Australia; ^2^Cognosco, Anexa Veterinary Services, Morrinsville, New Zealand; ^3^College of Public Health, Medical and Veterinary Sciences, James Cook University, Townsville, QLD, Australia

**Keywords:** ultrafiltration, internal standard, pharmacokinetics, moxidectin, carprofen, sheep

## Abstract

*In vivo* ultrafiltration has been used in veterinary pharmacokinetics since the early 2000’s as an improvement on the tissue cage model which enables sampling of fluids from extra-circulatory compartments. Variability in analyte recovery from ultrafiltration samples, due to membrane fouling or tissue inflammation, has been a concern for this technique. Internal standards may be used to scale or verify the unknown result, such as is common in analytical extractions and *in vivo* microdialysis. Eight merino sheep were implanted with subcutaneous tissue cages and 2 weeks prior to the initiation of the study the sheep were injected with 0.2 mg/kg moxidectin subcutaneously. On the day of the study ultrafiltration probes were inserted subcutaneously. At time zero 4 mg/kg of carprofen was injected intravenously. Plasma, tissue cage, and ultrafiltration samples were taken 30 min before and 0.5, 1, 2, 3, 4, 5, 7, 24, 36, 48, 72 h after dosing. Carprofen and moxidectin concentrations were measured by LC–MS/MS. Pharmacokinetic parameters were estimated using Monolix for both the carprofen concentrations and the moxidectin corrected carprofen concentrations. The ultrafiltration probes failed to consistently produce enough sample volume to analyse. Moxidectin concentrations in the plasma and tissue cage fluid were stable throughout the 72 h sampling window. Moxidectin proved to be suitable as an *in vivo* internal standard for pharmacokinetic research using, tissue cages, plasma sampling and ultrafiltration probes, but the application of ultrafiltration techniques requires refinement.

## 1 Introduction

*In vivo* ultrafiltration has been used in veterinary pharmacological research since at least the early 2000’s (1) although adoption of the method has been slow since its early description for drug distribution studies in 1992 (2). *In vivo* ultrafiltration is viewed as an improved model for tissue fluid sampling when compared to tissue cage models (3). An ultrafiltration probe, consisting of multiple loops of a membrane that is porous to molecules less than 30,000 Da, is inserted subcutaneously and connected to a collection vial under negative pressure (vacutainer). The collection vial is replaced at nominated timepoints to give multiple samples. The concentration of the analyte is determined and is regarded to be the average concentration of the sampling period, usually plotted as the mid-point of the sample period. This methodology was developed for glucose monitoring by Janle et al. and reported in 1987 (4).

Temperature, pressure differential, the chosen membrane material and the surface area of membrane affect the flux of analyte across the membrane (2). In addition to these factors fouling has been acknowledged to change the flux across the membrane (5). Imsilp et al. (6) reported inflammatory responses to intramuscular implantation of polyacrylonitrile ultrafiltration probes in sheep, the concern being that inflammation may change the recovery of target analytes. Bungay et al. (7), developed a model to account for the inflammation induced by the implantation of microdialysis probes, which are constructed of the same material as ultrafiltration probes, in neural tissue. The model predicted that the inflammation would lead to an underestimation of analyte concentrations. Previous uses of ultrafiltration probes for pharmacokinetic sampling have not described methods for incorporating an internal control to compensate for these changes in analyte recovery.

In contrast to ultrafiltration, microdialysis techniques use a pumped perfusate that contains a known concentration of a compound as an internal standard. This compound along with the target analyte is measured, and the change in the internal standard is used to calculate the relative recovery of target analyte. Linhares and Kissinger (8) compared ultrafiltration and microdialysis simultaneously for pharmacokinetic measurements of theophylline in a small number (*n* = 3) of rats, the concentrations obtained between the two techniques differed but the slope and shape of the concentration time graph was similar.

Ideal internal standards for microdialysis, as for other analytical techniques such as liquid chromatography, have similar physicochemical properties to the compound being studied. For microdialysis, the internal standard should diffuse through the membrane in a similar way to the compound of interest and be metabolised by similar pathways in the tissue (9). Some authors have employed radioisotopes to achieve this (10). As the internal standard is pumped into the membrane and tissue at a constant rate, in microdialysis the internal standard must have a short elimination half-life so that a steady state can be reached quickly.

A systemically administered drug which achieves steady-state concentration could provide an internal control for ultrafiltration sampling. At steady state concentrations the internal standard would be expected to be at a consistent concentration in any given tissue, thus changes in the concentration particularly decreases would indicate a potential change in the recovery of the analyte of interest in the ultrafiltrate. The relative change in the internal standard could be used to correct for the change in recovery.

Moxidectin is an antiparasitic macrocyclic lactone, commonly used in veterinary medicine for the treatment of intestinal nematodes and external parasites such as lice. In addition, it has activity against heartworm microfiliria (*Dirofilaria immitis*) in dogs. The pharmacokinetics of moxidectin in sheep have been described (11). Due to its long half-life of ~18 and 19 days in sheep and dogs, respectively, it can be dosed monthly for heartworm prevention (12) or provide residual activity against intestinal parasites such as *Haemonchus contortus.* Pseudo-steady state plasma concentrations, relative to short acting drugs, can be achieved after single or infrequent dosing. These characteristics make it a potential candidate for use an *in vivo* internal standard.

Carprofen is a non-steroidal anti-inflammatory drug (NSAID) widely used in veterinary medicine for the treatment of pain and inflammation. It has been widely used in pharmacological studies in multiple species with both tissue cage and ultrafiltration models, including description of its pharmacokinetics in sheep (3, 13–17).

In our previous work in sheep (16) simultaneous samples were collected, from five sizes of tissue cages and from blood, following carprofen dosing. The surface area to volume ratio differed between the tissue cage sizes and a model was constructed to simultaneously fit the pharmacokinetics for plasma and all tissue cages. A linear relationship was used to describe the change in microconstants for flux between the central compartment and the tissue cages. The surface area to volume ratio was demonstrated to affect the pharmacokinetic results in the tissue cages. Ultrafiltration probes have minimal volume and an extremely large diffusable surface area and therefore should represent the gold standard for measuring drugs in tissue fluid as the SA:V is practically infinite.

The aims of this study are two-fold. First, we aim to acquire simultaneous data from a tissue cage model and ultrafiltration probes, allowing contrast or comparison and possible validation of the different experimental models. Second, this study aims to investigate the viability of using moxidectin as an *in vivo* internal standard for tissue fluid (ultrafiltration and/or tissue cage) sampling. Our hypotheses are, ultrafiltration data will resemble plasma pharmacokinetics more than tissue cage data does and moxidectin will provide a pseudo-steady state concentration for the duration of the experiment and changes in moxidectin concentration will reflect changes in carprofen recovery.

## 2 Materials and methods

Animal work was approved by the University of Melbourne Faculty of Veterinary and Agricultural Sciences Animal Ethics Committee (UoM 2,015,111).

Eight merino wethers, approximately 18 months old and ranging from 42–51.5 kg, were enrolled. Each wether was determined to be healthy by veterinary clinical examination and routine haematological and biochemical testing prior to enrolment. All sheep were housed in a corrugated iron shed on slatted floors with water supplied *ad libitum*. Pellets (Sheep & Cattle Rumevite, Townsville QLD Australia) and lucerne chaff were provided daily. Ventilation was provided by passive air movement through doors and windows, and experiments were conducted between October and November 2020 in Werribee, Victoria, Australia (18).

Animals were anaesthetised and two tissue cages (6 cm and 10 cm length) were inserted under the skin on one side of the neck three weeks prior to the experiment, as previously described (16, 18), with the addition of a local anaesthetic field block being placed around the surgical site with 0.75% ropivacaine. The side of neck into which tissue cages were implanted was alternated so that 4 sheep had the left side implanted and 4 the right side.

Fourteen days prior to the experiment 0.2 mg/kg of moxidectin (Cydectin, Virbac, Milperra NSW Australia) was injected subcutaneously into a hind limb.

On the morning of experiment a cephalic vein was catheterised with an 18 g IV catheter (Jelco Optiva, Smiths Medical Macquarie Park NSW) and an injection port was attached. The port and catheter were secured with tape and flushed with heparinised saline after each sample. The side of the neck without tissue cages was clipped and aseptically prepared with iodine scrub. Two blebs of 2% lignocaine were injected under the skin and a stab incision made through the bleb. A sterile metal introducer trocar was tunnelled subcutaneously from the incision ventrally, the ultrafiltration probe was inserted inside the trocar, the trocar was then removed while maintaining the probe in place. The probe was secured to the sheep by placing butterfly tape wings around the exposed tubing and stapling the wings to the skin with skin staples. The tubing was connected to a double ended needle mounted on a vacutainer sampling bell. A sample vial with negative pressure was placed on the end of the needle.

At time zero, 4 mg/kg of carprofen was injected intravenously into the cephalic vein contralateral to the intravenous catheter. Samples of blood and tissue cage fluid were obtained at timepoints, −0.5, 0.5, 1, 2, 3, 4, 5, 7, 24, 36, 48, 72 h. The sample vial connected to each of the ultrafiltration probes was changed at these same timepoints. Samples of blood were obtained via the cephalic catheter, 1.5 mL of blood was withdrawn and discarded before a 4 mL sample was obtained, the catheter was flushed with heparinised saline. Tissue cage fluid was obtained by percutaneous puncture of the cage with a 20 ga hypodermic needle, analgesia was provided by the Coolsense device (18). All samples were collected into or transferred into sample vials containing lithium heparin.

Samples were stored at 4°C prior to centrifugation, the plasma (blood) or sediment free (tissue cage) fluid was decanted into 1.5 mL microcentrifuge tubes and stored at −80°C until analysis (≤21 days). Carprofen and Moxidectin have been shown to be stable in canine and human plasma, respectively at −80°C (19, 20).

At the end of the in-life phase the sheep were euthanised with pentobarbitone IV through the cephalic catheter. Tissue from around the ultrafiltration probe collected and submitted for histopathological examination.

### 2.1 Analytical method

Samples were subjected to separate carprofen and moxidectin LCMS analysis. Carprofen analysis was as previously described (16) with the following refinements; sample preparation was simplified to 100 μL of sample in addition to 400 μL of Meclofenamic Acid Internal Standard Working Solution being added to Ostro Pass-through plates (Waters Australia Rydalmere, NSW). Acetonitrile (ACN) was replaced with 50: 50 methanol: ACN as mobile phase B. Mobile phase A was MilliQ water without addition of buffer and the injection volume was reduced to 5 μL. The standard curve was extended to 100 μg/mL. The assay was partially validated for concentrations between 0.25 and 100 μg/mL. Spiked plasma was analysed at each point of the standard curve with 6 samples to calculate inter-sample CV. Additionally intra-assay CV was calculated at 50, 10, 1 and 0.25 μg/mL. All validation was done in a single day, inter-day variability was not assessed. Inter-assay variability was 1.9%–4.7% and intra-assay variability was 0.4%–2.3%.

A moxidectin method was developed and partially validated. Sample preparation for moxidectin analysis was; 400 μL of sample was mixed with 1,000 μL of Abamectin Internal Standard Working Solution (500 ng/mL ACN solvent) in a microcentrifuge tube. This was centrifuged and decanted. The supernatant was evaporated until dry in a Speedvac (Environmental Speedvac Savant, United States) on medium setting. The samples were reconstituted with 200 μL of ACN before transferring to 96-well plates for analysis. A 20 μL injection was made by the autosampler into a Shimadzu LCMS/MS system fitted with a C18 column as previously described (16), the column oven was held at 50°C. The liquid chromatography program began at 30% organic mobile phase (Isopropyl Alcohol:ACN, 75:25) rising to 90% organic at 5 min, the mobile phase was rapidly switched to 95% aqueous (MilliQ water) before returning to the starting conditions for 2 min. The nebulising gas, heating gas and drying gas were set to 2, 10 and 10 L/m, respectively. The interface temperature, DL temperature and heating block were set to 375, 250, and 400°C. Mass spectrometry was carried out in MRM mode with negative electro spray ionisation. Abamectin m/z 871.6 → 229.2 and 565.5 with collision energies of 27 and 28 eV, respectively. Moxidectin m/z was monitored 638.3 → 602.4, 236.2, and 247.1 with CE 20, 27, 26 eV, respectively. The assay was partially validated for concentrations between 10 ng/mL and 0.25 ng/mL. Intra-assay CV was calculated for 10, 5, 2.5, 1, 0.5, 0.25 ng/mL. Inter-assay variability was 5.3%–18.5%, intra-assay variability was 2.1%–5.9%.

Plasma spikes of moxidectin or carprofen were included in each analytical run as quality control. The results of the samples were adjusted based on the ratio of the measured concentration and the known concentration of the spiked plasma.

Since the sample volumes obtained from the ultrafiltration probes were generally low, the LCMS method was modified for small volumes; 60 μL of sample was combined with 150 μL of abamectin ISWS for moxidectin analysis, 100 or 20 μL of sample was combined with 400 or 80 μL of MFA ISWS depending on available sample volume. The rest of the sample preparation followed as above with volumes adjusted to maintain ratios. In most cases the entire sample was used thus preventing repeat analysis of samples.

### 2.2 Statistical methods

Pharmacokinetic analysis was performed in Monolix (2023R1, SimulationsPlus) using a custom model as previously described (16), the tissue cage concentrations were driven by the central compartment with the length of the cage in centimeters used as the regressor value. The model was run on both raw carprofen concentrations and on the corrected carprofen concentration. Confidence intervals for the parameters estimated by Monolix were generated using the Rsmlx package in R (21).

## 3 Results

Valid plasma carprofen results were obtained for all timepoints in all sheep except for sheep 6 at 36 h. Plasma moxidectin was not detectable in sheep 1 at −0.5 and sheep 6 at 72 h. In the tissue cages carprofen could not be measured in sheep 2 at 5 h in the 6 cm cage and sheep 4 at 1 h in the 10 cm cage. Moxidectin could not be measured in 6 samples from the tissues cages in sheep 6 and 8.

Ultrafiltration yielded 74 samples from which carprofen (*n* = 71), moxidectin (*n* = 19) or both (*n* = 16) could be quantified. The carprofen concentrations were generally low with 66 samples having concentrations below 1 ng/mL.

Moxidectin concentrations in plasma had little variation within individual sheep over the sampling time period with mean (CV) plasma moxidectin of 8.55–8.57 (0.05–0.15) ng/mL. Individual cage within subject mean moxidectin concentration in the tissue cages was 8.25–8.58 ng/mL with CV ranging from 0.03%–14%.

Carprofen concentrations were corrected when a valid concurrent moxidectin result was available by multiplying the carprofen result by the mean moxidectin concentration within the respective tissue and sheep divided by the moxidectin result.

### 3.1 Pharmacokinetics

Pharmacokinetic estimation was performed on the plasma and tissue cage data simultaneously, but insufficient valid results were available from the ultrafiltration probes to perform analysis on these data. A continuous co-variate was included in the model to describe the change in flux between the tissue cage and the central compartment due to the tissue cage size.

**Figure 1:**
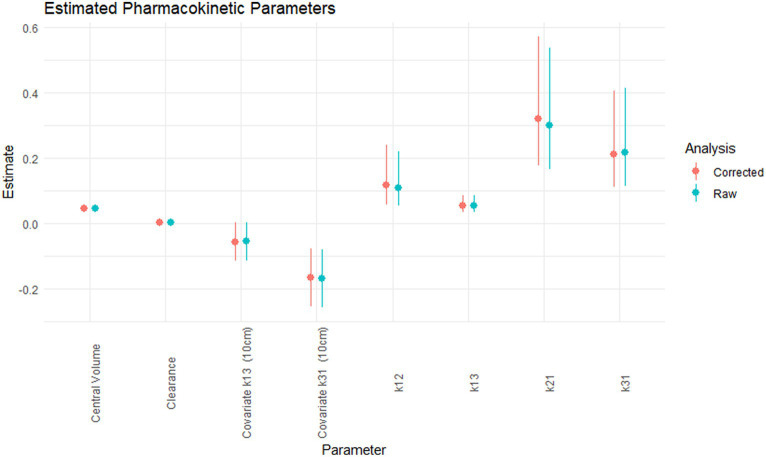
The estimated pharmacokinetic parameters; central volume (L/kg), clearance (L/kg.h), covariate for k13 for 10 cm cage (h^−1^), covariate for k31 for 10 cm cage (h^−1^), k12 (h^−1^), k13 (h^−1^), k21 (h^−1^), k31 (h^−1^) for the raw (green) and corrected carprofen.

The pharmacokinetic parameters estimated for the raw and corrected carprofen concentrations, and the 95% confidence interval are shown in Figure 1. Correction of the carprofen concentrations using moxidectin concentrations did not alter the estimated values of the pharmacokinetic parameters. The degree of uncertainty for these values was unaffected as shown by the 95% confidence intervals. The precise estimates are available in the Supplementary Table S1, but are not reported, as discussed below.

### 3.2 Histopathology

**Figure 2:**
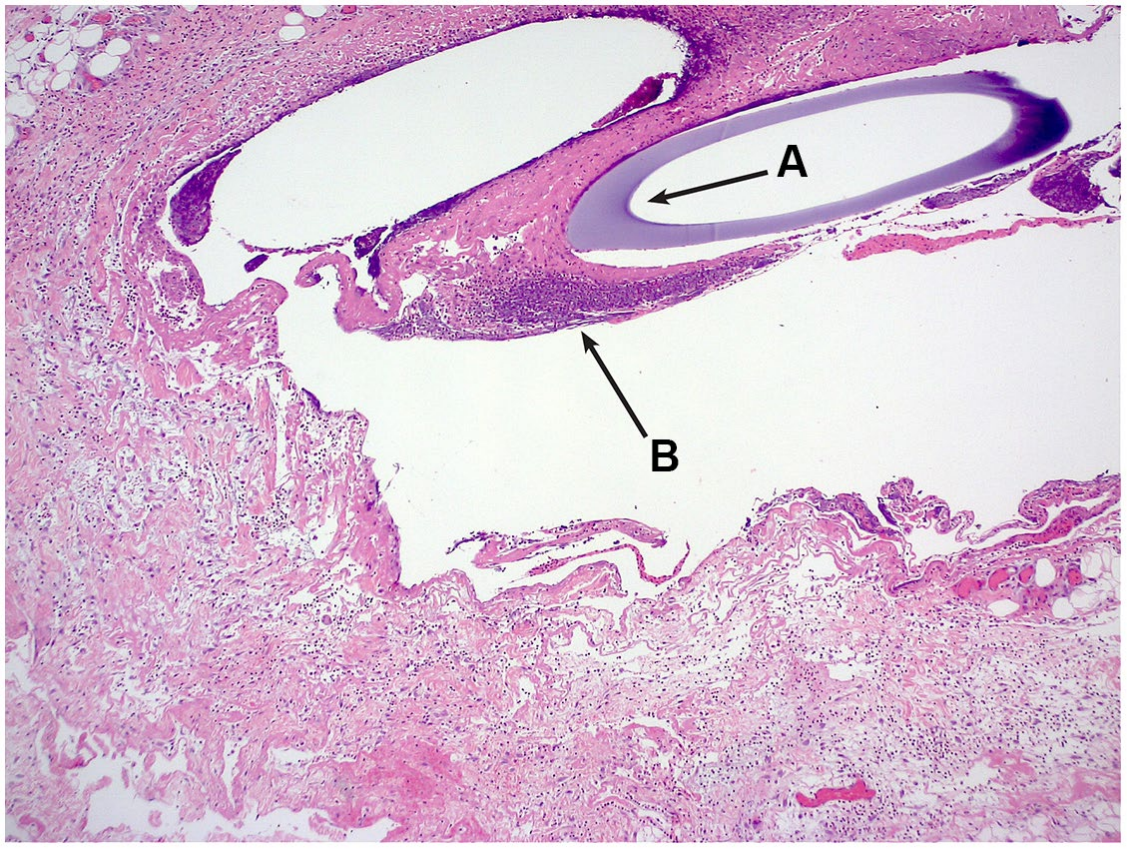
H&E photomicrograph, the cross-section of the ultrafiltration probe (A) can be seen, surrounded by inflammatory infiltrate (B).

A single ultrafiltration probe was recovered post-mortem *in situ* and submitted for histology. Six sections of skin to the level of the subcutaneous adipose tissue and skeletal muscles were examined. All sections reveal comparable changes but the changes were more prominent in sections from the distal part of the string implant. Located within the deep subcutis and forming a mantle around cavitated spaces which on occasion contain the cross section of a basophilic tubing was a moderate, mixed inflammatory infiltrate. This infiltrate was dominated by macrophages including multinucleated giant cells, neutrophils and eosinophils along with fewer lymphocytes and rare plasma cells. Also noted within these inflamed areas were free erythrocytes as well as accumulations of amorphous to fibrillar eosinophilic material with the appearance of fibrin and necrosis. There were marginal accumulations of fibroblasts, and small-calibre vessels and collagen fibres (granulation tissue). No bacteria were identified on gram staining. The morphological diagnosis was of Moderate, chronic, multifocal, pyogranulomatous panniculitis with an intralesional foreign body (Figure 2).

## 4 Discussion

Moxidectin concentrations measured in the plasma and tissue cage fluid showed little variability throughout the sampling period. This demonstrates that moxidectin is suitable as an *in vivo* internal standard for sampling periods of up to 72 h in sheep when given 14 days before the planned first sample. The stability of moxidectin in tissue cage fluid suggests that the removal of sample fluid did not directly influence the pharmacokinetic values obtained. Moxidectin is unlikely to be suitable for studies longer than 72 h as the concentrations would be expected to decrease by >10% with a half-life of 18 days. To the authors knowledge this is the first use of an internal standard for the validation of ultrafiltration and/or tissue cages and provides information for the future use of internal standards in these sampling methodologies.

The pharmacokinetic values did not differ in clinically or statistically relevant (22) amounts between the raw carprofen and carprofen adjusted for moxidectin concentrations. The volume of the central compartment calculated on this dataset was approximately 50% smaller than our previous calculations, clearance was reduced (16). This resulted in an estimated half-life of 19.5 h compared to 27.2 h in the previous dataset. The rate constants k21 for distribution into the peripheral compartment was larger while k13, and k31 representing flux of carprofen into and out of the tissue cages were smaller. These differences may be artefactual from the analytical phase as large corrections were required based on the standard curves included in the runs. Additionally the present dataset only contains 2 tissue cage sizes as opposed to 5 in our previous work. The pharmacokinetic parameter estimates in the current study allow comparisons within this study, but are unlikely to be as externally valid as in our previous report (16) and therefore are not relied on; the estimation of pharmacokinetic indices was not an objective of this study.

There are potential implications of using moxidectin as an *in vivo* internal standard when evaluating the pharmacokinetics of other drugs, such as carprofen. Drug–drug interactions between carprofen and moxidectin are possible in sheep, with potential for the pharmacokinetics of one or both drugs to be altered. However, no reports of interactions could be found in the literature for any species. These two drugs are likely to be given coincidentally in dogs, with moxidectin used for heartworm prevention and carprofen a common anti-inflammatory in general practice. No reports of adverse events or interactions were found. To fully validate the use of moxidectin as an *in vivo* internal standard, cross-over studies with the drug of interest could be performed to check for interactions in plasma. Similarly, it would be valuable to evaluate *ex vivo* and analytical interactions that might influence derived sample-drug concentration results.

Recovery sample volume of ultrafiltrate from probes was very poor with only one probe consistently producing sample volumes of 100 μL or more. This necessitated the LCMS methodology to be adapted to small volumes and prevented re-analysis of samples, as no additional sample was available. In the current study the probes were inserted subcutaneously using methodology previously described on the same day as the sampling period began. In contrast, some previous publications have inserted the probes 24 h or more ahead of sampling. Our decision to insert probes on Day One was determined from pragmatic needs, as in both group-housed sheep and for sheep in individual pens, the neighbouring animals attempted to chew the probe tubing. Same-day insertion was also intended to minimise the effect of the inflammatory obstruction of probe membranes previously described (6). Sample volumes from the probes did not increase substantially over the 72 h sampling period, suggesting that the timing of insertion did not influence sample volume recovery.

In contrast to their use by other authors (4) the stab incisions through which the probes were inserted were not closed in this study. This may have led to an air gap between the probe and subcutaneous tissues, which could be maintained by the continued entry of air from the wound. Future work may benefit from sealing the insertion wound. Species differences may contribute to the amount of ultrafiltrate that can obtained. The early work of Janle stated that subcutaneous probes produced 0.5–1 mL of ultrafiltrate per day. However, Plummer et al. (23) also report low sample volumes (maximum of 1 mL per 4–12 h) from ultrafiltration probes in merino sheep. Additionally, Plummer et al. report a high failure and complication rate of UF probes in sheep, similar to our experience in the current study. Similar experiences have been reported in calves. Hauschild et al. (24) reported that drug concentrations of pradofloxacin in ultrafiltrate sample were below the LOD when the sample volume was low, the authors infer there was insufficient vacuum to drive the drug molecules across the membrane, this phenomenon was also observed in our study. Advances in analytical technology allowing smaller samples to be utilised may overcome some of these barriers but will not overcome the poor recovery seen with low flow rates as described by Hauschild and this paper.

In this work 4 mL evacuated tubes were used to apply the vacuum pressure and collect the sample. This similar to other authors (4, 25), although flow rates in those papers were low. The use of larger evacuated tubes, i.e., 10 mL would be expected to apply a greater vacuum pressure and increase the flow rate of the ultrafiltrate.

Cooke et al. (26) overcame some of these difficulties by utilising a hybrid method, by implanting a bundle of microdialaysis fibres (*n* = 176) subcutaneously, with both ends accessible. Samples were obtained by flushing the bundle intermittently thus they were able to forgo the perfusate pump system of traditional microdialysis and ensure the sample volume was sufficent for analysis by HPLC. The equilibration of the fibre bundle to the surrounding environment was reported to be 5 min allowing dense pharmacokinetic sampling to occur. Despite the apparent success of this technique, no other reports of its use were found. This technique may warrant further use given the reported difficulties of ultrafiltration and the limitations of microdialysis equipment.

The limited reported use of ultrafiltration probes for pharmacological studies outside laboratory rats gives rise to the possibility of publication bias, the work by Plummer et al. (23), Bidgood and Papich (1), Messenger et al. (3) and this paper were part of graduate research programs, thus increasing the likelihood of submission of unfavourable results as publication is often a prerequisite for speciality (27, 28). Commercially funded work using ultrafiltration probes that did not produce sufficient volumes for analysis may not have been submitted or accepted for publication (29).

The histopathological findings on the recovered probe were consistent with those reported by Imsilp et al. (6) with granulomatous inflammation without evidence of bacterial infection. This suggests the probes suffered from biofouling and encapsulation as described by Wisniewski et al. (30). There appears to be marked species differences in the reaction to ultrafiltration probes with Underwood reporting mild inflammation in line with suture material reaction when ultrafiltration probes were inserted in equine lamella tissue (25).

Given the consistent plasma and tissue cage fluid concentrations of moxidectin, it would be expected that moxidectin would be recovered in concentrations of a similar order and variability from the ultrafiltration probes if they were functioning correctly. The use of moxidectin as an *in vivo* internal standard for pharmacokinetic studies would allow researchers to detect changes in recovery and correct for them post-hoc. This should lead to more reliable and robust results.

## 5 Conclusion

Subcutaneously implanted tissue cages are a more reliable model for obtaining non-central compartment samples than commercially available ultrafiltration probes. Unfortunately, tissue cage-derived samples are less likely to represent true physiological spaces than samples obtained by ultrafiltration (16).

Currently reported ultrafiltration probe techniques appear to be unsuitable for pharmacokinetic sampling. In agreement with prior work, we found that the method will require modification or refinement if it is to give consistent samples. Effective modifications and refinements have not been identified.

We were unable to accept or reject our first hypothesis as we could generate sufficient simulataneous data from the two methodologies for comparison.

This work showed that moxidectin is useful as an *in vivo* internal standard for pharmacokinetic studies. In sheep, a single dose proved useful for a duration of approximately 3 days when injected subcutaneously 2 weeks prior to the study commencement. Samples from both tissue cages and ultrafiltration probe studies illustrated this usefulness. Thus we can partially accept the second hypothesis, we did not observe changes in moxidectin concentration of sufficient magnitude to evaluate the second portion of the hypothesis.

Further work to characterize moxidectin as an *in vivo* internal standard in ultrafiltrate samples should await discovery of effective modifications and refinements to ultrafiltration sample techniques which enable reliable acquisition of samples.

## Data availability statement

The raw data supporting the conclusions of this article will be made available by the authors, without undue reservation.

## Ethics statement

The animal study was approved by University of Melbourne Faculty of Veterinary and Agricultural Sciences Animal Ethics Committee. The study was conducted in accordance with the local legislation and institutional requirements.

## Author contributions

RM: Data curation, Formal analysis, Investigation, Methodology, Project administration, Writing – original draft, Writing – review & editing. TW: Conceptualization, Investigation, Methodology, Project administration, Resources, Supervision, Writing – review & editing.

## References

[1] Bidgood T Papich MG. Plasma pharmacokinetics and tissue fluid concentrations of meropenem after intravenous and subcutaneous administration in dogs. Am J Vet Res. (2002) 63:1622–8. doi: 10.2460/ajvr.2002.63.1622, PMID: 12492274

[2] Linhares MC Kissinger PT. Capillary ultrafiltration: in vivo sampling probes for small molecules. Anal Chem. (1992) 64:2831–5. doi: 10.1021/ac00046a029, PMID: 1294008

[3] Messenger K Wofford J Papich M. Carprofen pharmacokinetics in plasma and in control and inflamed canine tissue fluid using in vivo ultrafiltration. J Vet Pharmacol Ther. (2016) 39:32–9. doi: 10.1111/jvp.12233 25958925

[4] Janle-Swain E Van Vleet JF Ash SR. Use of a capillary filtrate collector for monitoring glucose in diabetics. ASAIO J. (1987) 33:336–40. 3675963

[5] Garrison KE Pasas SA Cooper JD Davies MI. A review of membrane sampling from biological tissues with applications in pharmacokinetics, metabolism and pharmacodynamics. Eur J Pharm Sci. (2002) 17:1–12. doi: 10.1016/S0928-0987(02)00149-5 12356415

[6] Imsilp K Whittem T Koritz GD Zachary JF Schaeffer DJ. Inflammatory response to intramuscular implantation of polyacrylonitrile ultrafiltration probes in sheep. Vet Res. (2000) 31:623–34. doi: 10.1051/vetres:2000145 11129805

[7] Bungay PM Newton-Vinson P Isele W Garris PA Justice JB Jr. Microdialysis of dopamine interpreted with quantitative model incorporating probe implantation trauma. J Neurochem. (2003) 86:932–46. doi: 10.1046/j.1471-4159.2003.01904.x, PMID: 12887691 PMC2386091

[8] Linhares MC Kissinger PT. Pharmacokinetic monitoring in subcutaneous tissue using in vivo capillary ultrafiltration probes. Pharm Res. (1993) 10:598–602. doi: 10.1023/A:1018914522749 8483845

[9] de Lange EC. Recovery and calibration techniques: toward quantitative microdialysis In: Müller M Microdialysis in drug development. New York: Springer (2012). 13–33.

[10] MacLean DA Ettinger SM Sinoway LI Lanoue KF. Determination of muscle-specific glucose flux using radioactive stereoisomers and microdialysis. Am J Physiol Endocrinol Metab. (2001) 280:E187–92. doi: 10.1152/ajpendo.2001.280.1.E187, PMID: 11120673

[11] Pérez R Núñez MJ Palma C Riquelme J Arboix M. Plasma disposition kinetics of moxidectin after subcutaneous administration to pregnant sheep. J Vet Pharmacol Ther. (2014) 37:550–5. doi: 10.1111/jvp.12127, PMID: 24731163

[12] Mealey KL. Canine ABCB1 and macrocyclic lactones: heartworm prevention and pharmacogenetics. Vet Parasitol. (2008) 158:215–22. doi: 10.1016/j.vetpar.2008.09.009, PMID: 18922637

[13] Brentnall C Cheng Z Mckellar QA Lees P. Influence of oxytetracycline on carprofen pharmacodynamics and pharmacokinetics in calves. J Vet Pharmacol Ther. (2013) 36:320–8. doi: 10.1111/jvp.12000, PMID: 22913421

[14] Cheng Z Nolan A McKellar Q. Anti-inflammatory effects of carprofen, carprofen enantiomers, and NG-nitro-L-arginine methyl ester in sheep. Am J Vet Res. (2002) 63:782–8. doi: 10.2460/ajvr.2002.63.782 12061520

[15] Lees P Delatour P Benoit E Foster AP. Pharmacokinetics of carprofen enantiomers in the horse. Acta Vet Scand Suppl. (1991) 87:249–51.

[16] Munn R Whittem T Woodward AP. The surface area to volume ratio changes the pharmacokinetic and pharmacodynamic parameters in the subcutaneous tissue cage model: as illustrated by carprofen in sheep. Front Vet Sci. (2022) 9:801. doi: 10.3389/fvets.2022.905797 PMC9284023 35847628

[17] Welsh EM Baxter P Nolan AM. Pharmacokinetics of carprofen administered intravenously to sheep. Res Vet Sci. (1992) 53:264–6. doi: 10.1016/0034-5288(92)90123-J 1439219

[18] Munn R Woodward A Beths T Whittem T. Observations on the use of a pain numbing device for repetitive percutaneous sampling in sheep. Aust Vet J. (2021) 99:445–8. doi: 10.1111/avj.13104, PMID: 34180048

[19] Shuttleworth JR Behrens KN Biggo MR Horne RL Cox S Lakritz J. Effect of storage duration on carprofen concentration measurements in dog plasma. Vet Med Sci. (2023) 9:2022–5. doi: 10.1002/vms3.1215, PMID: 37471576 PMC10508557

[20] Chhonker YS Sleightholm RL Murry DJ. Bioanalytical method development and validation of moxidectin in plasma by LC–MS/MS: application to in vitro metabolism. Biomed Chromatogr. (2019) 33:e4389. doi: 10.1002/bmc.4389 30238696

[21] Lavielle M., Rsmlx: R Speaks 'Monolix'. Repository CRAN. Eds. Mihaljevic F, Lavielle M, Chauvin J., Pinaud C. (2022). Available at: https://monolix.lixoft.com/rsmlx/

[22] Cumming G Finch S. Inference by eye: confidence intervals and how to read pictures of data. Am Psychol. (2005) 60:170–80. doi: 10.1037/0003-066X.60.2.170, PMID: 15740449

[23] Plummer C White PJ Kimble B Govendir M van der Saag D. Preliminary investigation into a novel sustained-release formulation of meloxicam in sheep (*Ovis aries*)—pharmacokinetic profile. Animals. (2021) 11:2484. doi: 10.3390/ani11092484, PMID: 34573450 PMC8466480

[24] Hauschild G Rohn K Engelhardt E Sager M Hardes J Gosheger G. Pharmacokinetic study on pradofloxacin in the dog – comparison of serum analysis, ultrafiltration and tissue sampling after oral administration. BMC Vet Res. (2013) 9:32. doi: 10.1186/1746-6148-9-32, PMID: 23410255 PMC3598979

[25] Underwood C Collins SN van Eps AW Allavena RE Medina-Torres CE Pollitt CC. Ultrafiltration of equine digital lamellar tissue. Vet J. (2014) 202:314–22. doi: 10.1016/j.tvjl.2014.05.007, PMID: 25439438

[26] Cooke I Bevill R Koritz G. Pharmacokinetics of penicillin G in plasma and interstitial fluid collected with dialysis fiber bundles in sheep. Vet Res. (1996) 27:147–59. PMID: 8721294

[27] Wood W McCollum J Kukreja P Vetter IL Morgan CJ Hossein Zadeh Maleki A. Graduate medical education scholarly activities initiatives: a systematic review and meta-analysis. BMC Med Educ. (2018) 18:318. doi: 10.1186/s12909-018-1407-8, PMID: 30577779 PMC6303993

[28] The Australian and New Zealand College of veterinary scientists fellowship candidate handbook. (2023). Available at: https://ripehosting.blob.core.windows.net/anzcvs-dev-media/42753/20230724-fellowship-candidate-handbook-2023-finalised1.pdf

[29] Thornton A Lee P. Publication bias in meta-analysis: its causes and consequences. J Clin Epidemiol. (2000) 53:207–16. doi: 10.1016/S0895-4356(99)00161-4, PMID: 10729693

[30] Wisniewski N Klitzman B Miller B Reichert WM. Decreased analyte transport through implanted membranes: differentiation of biofouling from tissue effects. J Biomed Mater Res. (2001) 57:513–21. doi: 10.1002/1097-4636(20011215)57:4<513::AID-JBM1197>3.0.CO;2-E, PMID: 11553881

