# Supplementary material for: Moxidectin is a candidate for use as an in vivo internal standard in pharmacokinetic studies, as demonstrated with use in simultaneous tissue cage and ultrafiltration fluid collection
Source: Front Vet Sci. 2024 Jan 16;11:1332974. doi: 10.3389/fvets.2024.1332974 (PMC10824831; doi:10.3389/fvets.2024.1332974)
Supplement: Supplementary file 1 [file Table_1.DOCX]

Supplementary

| **Raw Analysis** | **Estimate** | | **S.E.** | **R.S.E.(%)** |
| --- | --- | --- | --- | --- |
| **Fixed Effects** | | | | |
| Central Volume (L/kg) | 0.045 | | 0.0021 | 4.69 |
| k12 (h^-1^) | 0.11 | | 0.044 | 40.7 |
| k21 (h^-1^) | 0.3 | | 0.096 | 32.2 |
| Clearance (L/h.kg) | 0.0016 | | 0.000088 | 5.54 |
| k31 (h^-1^) | 0.22 | | 0.078 | 35.8 |
| Covariate for k31 for Cage Size 6 cm (h^-1^) | 0* | |  |  |
| Covariate for k31 for Cage Size 10 cm (h^-1^) | -0.17 | | 0.045 | 26.5 |
| k13 (h^-1^) | 0.052 | | 0.013 | 25.3 |
| Covariate for k13 for Cage Size 6 cm (h^-1^) | 0* | |  |  |
| Covariate for k13 for Cage Size 10 cm (h^-1^) | -0.056 | | 0.03 | 54.0 |
| **Standard Deviation of the Random Effects** | | | | |
|  | **Value** | **C.V.(%)** | **S.E.** | **R.S.E.(%)** |
| Volume (L/kg) | 0.000091 | 0.0091 | 16.62 | 1.82e+7 |
| k12 (h^-1^) | 0.42 | 43.73 | 0.18 | 42.8 |
| k21 (h^-1^) | 0.00052 | 0.052 | 141.63 | 2.72e+7 |
| Clearance | 0.13 | 13.48 | 0.043 | 31.7 |
|  |  |  |  |  |
| **Moxidectin Corrected** |  | |  | |
|  | **Estimate** | | **S.E.** | **R.S.E.(%)** |
| **Fixed Effects** | | | | |
| Central Volume (L/kg) | 0.045 | | 0.0021 | 4.80 |
| k12 (h^-1^) | 0.12 | | 0.048 | 40.9 |
| k21 (h^-1^) | 0.32 | | 0.1 | 31.8 |
| Clearance (L/h.kg) | 0.0016 | | 0.000087 | 5.52 |
| k31 (h^-1^) | 0.21 | | 0.076 | 35.9 |
| Covariate for k31 for Cage Size 6 cm (h^-1^) | 0* | |  |  |
| Covariate for k31 for Cage Size 10 cm (h^-1^) | -0.17 | | 0.045 | 27.0 |
| k13 (h^-1^) | 0.053 | | 0.013 | 25.3 |
| Covariate for k13 for Cage Size 6 cm (h^-1^) | 0* | |  |  |
| Covariate for k13 for Cage Size 10 cm (h^-1^) | -0.057 | | 0.03 | 53.1 |
| **Standard Deviation of the Random Effects** | | | | |
|  | **Value** | **C.V.(%)** | **S.E.** | **R.S.E.(%)** |
| Volume (L/kg) | 0.00026 | 0.026 | 5.91 | 2.25e+6 |
| k12 (h^-1^) | 0.41 | 43.05 | 0.18 | 42.8 |
| k21 (h^-1^) | 0.00089 | 0.089 | 78.49 | 8.82e+6 |
| Clearance (L/h.kg) | 0.13 | 13.46 | 0.042 | 31.7 |
